# Bridging the Gap between Microsurgery and Supermicrosurgery: A Rat Study for 0.3 mm Vascular Anastomosis Comparing Robotic-assisted vs. Conventional Approach

**DOI:** 10.1055/a-2824-6437

**Published:** 2026-04-09

**Authors:** Tae Hyung Kim, Seohee Chae, Donggeon Kim, Taehyun Kim, Jin Geun Kwon, Changsik John Pak, Hyunsuk Peter Suh, Joon Pio Hong

**Affiliations:** 1 Department of Plastic and Reconstructive Surgery, Asan Medical Center, University of Ulsan College of Medicine, Seoul, South Korea

**Keywords:** supermicrosurgery, robotic microsurgery platform, Symani, 0.3 mm vessels

## Abstract

**Background:**

Supermicrosurgery involving vessels ≤0.3 mm remains among the most technically demanding procedures in reconstructive surgery. Robotic platforms have shown potential to enhance precision, but their feasibility at this extreme scale requires further validation.

**Methods:**

Apprentice surgeons (≤5 years' experience) performed rat femoral vessel (approximately 0.26–0.30 mm) anastomoses using both hand-sewn and robotic-assisted techniques across eight consecutive trials. Outcome measures included Structured Assessment of Robotic Microsurgical Skills (SARMS) scores, operative time, stitch count, and patency rate. Learning curves were assessed with cumulative summation (CUSUM) analysis, and anastomotic quality was evaluated using scanning electron microscopy (SEM).

**Results:**

Robotic-assisted anastomosis reached proficiency earlier than hand-sewn repair (4th versus 6th trial). Although initial operative time was longer for robotic cases (1,772 ± 120 seconds versus 1,355 ± 187 seconds, *p* = 0.013), times equalized by the 5th trial and decreased by 42% by the 8th (1,026 ± 58 seconds versus 1,023 ± 73 seconds, *p* = 0.959). Stitch counts were higher in later robotic trials (4.2 ± 0.6 versus 3.5 ± 0.5, *p* = 0.015). Both groups achieved 100% patency by the 4th trial. CUSUM and SEM analyses confirmed a steeper learning curve and superior anastomotic precision with robotic assistance.

**Conclusion:**

Robotic-assisted supermicrosurgery enables earlier proficiency, enhanced precision, and reproducible anastomotic quality compared with hand-sewn techniques. Despite longer initial times, robotic performance rapidly improved, achieving equivalent efficiency with greater technical control. These findings confirm the technical feasibility of robotic supermicrosurgery at the 0.3 mm scale and support its potential role in microsurgical and lymphatic reconstruction training and clinical practice.

## Introduction

Supermicrosurgery, defined as vascular anastomosis in vessels smaller than 0.8 mm, has expanded the frontier of reconstructive microsurgery by enabling procedures such as lymphaticovenous bypass and perforator-to-perforator flap transfers. 1 2 3 However, the execution of these delicate procedures remains limited to a small cohort of highly skilled surgeons due to the extreme precision required, steep learning curve, and fatigue associated with conventional manual techniques.

Recent technological advances have introduced robotic platforms specifically engineered for microsurgery. Among them, the Symani Surgical System offers wristed microinstruments, motion scaling, and tremor elimination—features theoretically well-suited for supermicrosurgical tasks. 3 4 5 Although early studies have reported the system's feasibility in anastomoses of conventional vessel sizes, its applicability in vessels at or below 0.3 mm remains largely unexamined. Such small-caliber vessels present unique challenges: minimal luminal space, fragility of the vessel wall, and increased susceptibility to thrombosis or leakage from even minor stitch misalignments. 4 6 7 8 9 10 11 12

Given the growing clinical interest in robotic-assisted supermicrosurgery, especially for lymphatic reconstruction involving vessel diameters of 0.3 mm or smaller, a critical question remains as to whether robotic platforms truly facilitate technical skill acquisition at such extreme microsurgical scales. 13 Although prior studies have confirmed the feasibility and safety of systems like the Symani Surgical System in both preclinical and clinical settings, few have directly compared the learning curves of robotic-assisted versus hand-sewn anastomoses at the supermicrosurgical level. 14 15 16

In a previous study, we demonstrated that robotic-assisted anastomoses in rat femoral arteries and veins enabled novice surgeons to achieve consistent patency with reduced vessel trauma. 6 In this follow-up investigation, we focused exclusively on vessels averaging 0.3 mm in diameter to simulate true supermicrosurgical conditions. By analyzing outcomes within a single-experience-level cohort and a uniform animal model, this study aims to provide critical insight into the precision, consistency, and feasibility of robotic supermicrosurgery at its technical limits.

## Methods

All animal experiments were conducted in accordance with protocols approved by the Institutional Animal Care and Use Committee (IACUC) of Asan Medical Center (University of Ulsan College of Medicine). This study focused on assessing robotic microsurgical performance in high-difficulty settings using 0.3 mm vessels.

A total of 32 Lewis rats underwent bilateral femoral artery and vein microanastomoses. Each animal received one hand-sewn and one robotic-assisted anastomosis per side, enabling direct intra-animal comparison. All procedures were performed under operative microscopy by microsurgical trainees (apprentice group) currently enrolled in a structured fellowship program. No expert or novice groups were included in this study to isolate robotic performance in an intermediate skill population.

Robotic-assisted procedures were conducted using the Symani Surgical System (Medical Microinstruments, Pisa, Italy), featuring motion scaling, tremor filtration, and wristed instrumentation. All anastomoses were performed under standardized conditions, and procedures were recorded for postoperative evaluation.

### Evaluation

1. Proficiency Assessment
Technical proficiency was assessed using a modified version of the Structured Assessment of Robotic Microsurgical Skills (SARMS), adapted for the robotic platform to incorporate domains of needle articulation and control. 17 Each participant completed eight consecutive trials, consisting of both hand-sewn and robotic procedures, resulting in a total of 16 anastomoses per operator.
Learning trajectories were analyzed using cumulative summation (CUSUM) of SARMS scores. 18 19 A predefined proficiency benchmark of 4.5 was uniformly applied to both groups. CUSUM plots were generated by subtracting this threshold from each observed score, enabling comparison of learning curves and identification of inflection points reflecting performance improvement between hand-sewn and robotic techniques. Additionally, raw SARMS scores were plotted to illustrate stabilization around the proficiency threshold.
2. Procedural Efficiency (Time per Single Vessel Anastomosis)
  Efficiency was evaluated by measuring total execution time, defined as the time taken to complete four sequential micro-movements: (1) needle grasping, (2) wall puncture, (3) stitch pull-through, and (4) thread cutting. CUSUM plots and normalized time deviation graphs were used to illustrate the progression of efficiency across trials.
3. Precision Evaluation
  To evaluate the precision of suturing, we quantified the number of stitches placed for each microvascular anastomosis. This measure was regarded as a surrogate marker of microanatomic precision, as a greater number of fine stitches per vessel may correlate to meticulous handling of the vessel wall and enhanced motor control of the operating surgeon.
4. Accuracy Evaluation
  Each anastomosis was scored on a three-point ordinal scale: 0 = complete failure; 1 = leakage present; 2 = watertight, fully successful. Accuracy scores were assigned separately for arterial and venous repairs.
5. Scanning Electron Microscopy (SEM) Evaluation
  For selected specimens, SEM was performed to evaluate endothelial integrity, stitch placement, and microtrauma. SEM findings were interpreted to compare structural precision between hand-sewn and robotic techniques.

### Statistical Analysis

All statistical analyses were performed using SPSS version 30.0 (IBM Corp., Armonk, NY). For comparison of outcomes across experience levels or phases, one-way analysis of variance (ANOVA) or paired *t* -tests were used as appropriate. A *p* -value <0.05 was considered statistically significant.

## Result

### Vessel Diameter

To ensure comparability, vessel diameters were analyzed across all groups, phases, and techniques. No statistically significant differences were observed in artery or vein diameter between the robotic-assisted and hand-sewn groups across all experience levels ( *p* > 0.05) ( Table 1 ).

**Table 1:** Comparison of vessel diameter between robot and hand techniques

Vessel	Robot Diameter (mean ± SD)	Hand Diameter (mean ± SD)	*p* -value
Artery	0.26 ± 0.05	0.26 ± 0.04	0.48
Vein	0.30 ± 0.07	0.30 ± 0.04	0.57

### Learning Curve Analysis

**Fig. 1:**
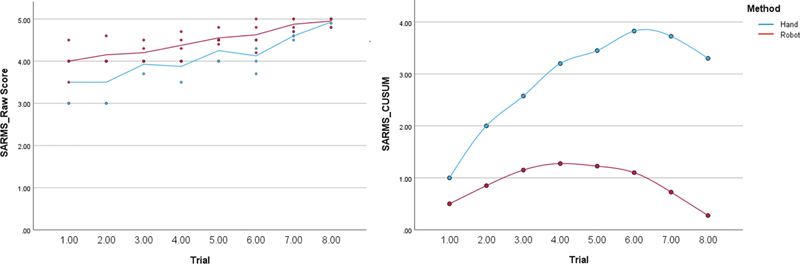
Modified Structured Assessment of Robotic Microsurgical Skills (SARMS) score learning curves. ( **A** ) Raw SARMS scores across trials. ( **B** ) Cumulative sum (CUSUM) analysis of SARMS scores.

**Fig. 2:**
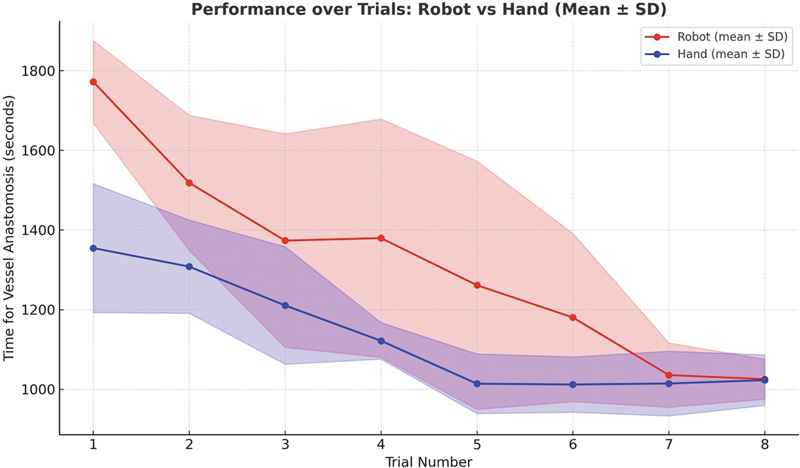
Total anastomosis time per single vessel anastomosis. Time frame evaluation for robotic-assisted (red) and hand-sewn (blue) procedures across all trials.

**Fig. 3:**
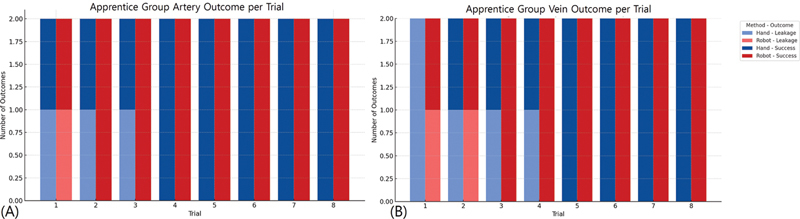
Anastomotic outcomes by vessel type. Stacked bar graphs showing technical success rates in arterial and venous anastomoses for each technique. Note that from the 2nd trial of the artery and 3rd trial of the vein robotic approach group showed 100% leakage-free anastomosis.

**Fig. 4:**
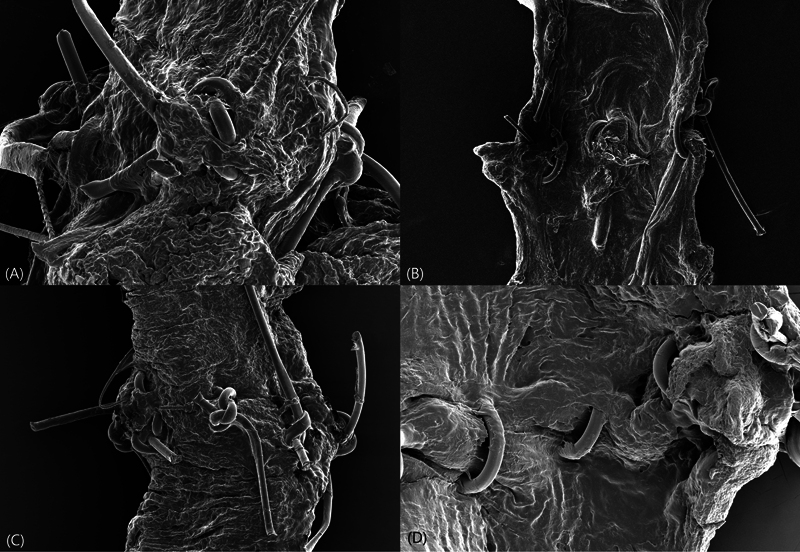
Scanning electron microscopy (SEM) of anastomosis sites. ( **A, B** ) Hand-sewn group. ( **C, D** ) Robotic-assisted group. SEM images illustrate superior stitch placement, vessel edge integrity, and microstructural outcomes for the robotic approach group.

1. SARMS-based Proficiency Development
Robotic-assisted SARMS scores were consistently higher than hand-sewn across trials, with significant differences at the 4th (4.38 ± 0.30 versus 3.88 ± 0.25, *p* = 0.044) and 7th trials (4.88 ± 0.15 versus 4.60 ± 0.08, *p* = 0.026). Both the groups showed progressive improvement, and scores converged by the 8th trial (4.95 ± 0.10 versus 4.93 ± 0.10, *p* = 0.730) ( Table 2 ). CUSUM analysis further indicated that the robotic group reached proficiency earlier (the 4th trial), whereas the hand-sewn group achieved stabilization later by the 6th trial ( Fig. 1 ).
2. Procedural Efficiency Measured by Time per Single Vessel Anastomosis
Regarding the artery, the robotic-assisted procedures initially required significantly more time at the first trial than hand-sewn techniques (1,772.0 ± 119.9 versus 1,354.5 ± 186.9 seconds, *p* = 0.013). However, from the 2nd trial there was no statistical difference although there was a tendency of longer time needed for the robotic group. Nevertheless, by the 7th trial, the robotic group demonstrated anastomosis time of 1,035.75 ± 93.27 seconds compared with the hand-sewn group of 1,014.50 ± 93.88 seconds, which was negligible ( *p* = 0.759). The anastomosis time for a single artery reduced by 42% over the trials.
The anastomosis time per vein revealed a significant longer time was needed for the hand-sewn group in the first two trials (2,040.2 ± 158.6 and 1,731.2 ± 222.6 seconds respectively) compared with the robotic-assisted group (1,471.8 ± 231.1 and 1,236.2 ± 162.4 seconds respectively) ( *p* = 0.009 and *p* = 0.013 respectively). The difference was negligible between the two groups by the 8th trial ( *p* = 0.512) ( Fig. 2 , Table 3 ).
3. Precision Evaluation Based on the Number of Stitch per Vessel
In the comparison of stitch numbers between hand-sewn (2.58 ± 0.51) and robotic-assisted groups (2.42 ± 0.51), there was no statistical significance for the first three trials of the arteries ( *p* = 0.436). However, from the 4th trial up to the last 8th trial, the robotic-assisted group (4.50 ± 0.51) demonstrated a significantly higher number of stitches compared with the hand-sewn group (4.20 ± 0.41) ( *p* = 0.048). These findings suggest that while stitch counts were comparable at the beginning, the robotic system enabled a greater number of stitches as the learning curve progressed. Vein stitch counts showed a similar tendency where there was no significant difference in the first three trials ( *p* = 0.083) but there were significantly higher number of stitches with robotics from the 4th trial ( *p* = 0.041) ( Table 4 ).
4. Accuracy Evaluation for Patent Anastomosis (Success/Leakage/Failure)
In both artery and vein anastomoses, the success rate reached 100% from 4th trial onward in both the robotic-assisted and hand-sewn groups ( Fig. 3 ). During the early phase (trials 1–3), leakage was observed in both methods, with higher incidence in the hand-sewn group. No failed anastomosis was reported in this phase.
5. SEM Evaluation
At the final (8th) trial, SEM was performed on microanastomoses completed on 0.3 mm vessels ( Fig. 4 ). The hand-sewn group demonstrated technical imperfections including loose stitches, tissue infolding, and localized microvascular wall damage. In contrast, the robotic-assisted anastomoses showed evenly spaced, tightly secured stitches with minimal microtearing and well-preserved vessel wall integrity. These findings suggest superior microscale precision and consistency with robotic assistance at peak proficiency.

**Table 2:** Comparison of modified SARMS scores between hand-sewn and robotic-assisted anastomosis across trials

Trial	Hand (mean ± SD)	Robot (mean ± SD)	*p* -value
1	3.50 ± 0.58	4.00 ± 0.41	0.212
2	3.50 ± 0.58	4.15 ± 0.30	0.108
3	3.92 ± 0.15	4.20 ± 0.24	0.114
4	3.88 ± 0.25	4.38 ± 0.30	0.044
5	4.25 ± 0.29	4.55 ± 0.17	0.136
6	4.12 ± 0.35	4.62 ± 0.35	0.09
7	4.60 ± 0.08	4.88 ± 0.15	0.026
8	4.93 ± 0.10	4.95 ± 0.10	0.73

Abbreviation: SARMS, Structured Assessment of Robotic Microsurgical Skills.

**Table 3:** Comparison of anastomosis time per vessel between hand-sewn and robotic-assisted groups

**(A) Arteries**			
**Trial**	**Hand (mean ± SD)**	**Robot (mean ± SD)**	***p*** **-value**
1	1,354.50 ± 186.85	1,772.00 ± 119.88	0.013
2	1,308.00 ± 135.29	1,518.50 ± 195.69	0.133
3	1,210.50 ± 170.38	1,373.25 ± 309.39	0.402
4	1,121.75 ± 53.09	1,379.50 ± 345.29	0.233
5	1,014.00 ± 86.58	1,261.25 ± 359.82	0.265
6	1,012.00 ± 80.29	1,180.25 ± 243.65	0.266
7	1,014.50 ± 93.88	1,035.75 ± 93.27	0.759
8	1,023.00 ± 73.38	1,025.50 ± 57.99	0.959
**(B) Veins**			
**Trial**	**Hand (mean ± SD)**	**Robot (mean ± SD)**	***p*** **-value**
1	2,040.2 ± 158.6	1,471.8 ± 231.1	0.009
2	1,731.2 ± 222.6	1,236.2 ± 162.4	0.013
3	1,494.8 ± 383.1	1,181.5 ± 119.3	0.202
4	1,591.0 ± 490.3	1,095.5 ± 65.0	0.136
5	1,511.8 ± 386.7	1,118.2 ± 162.9	0.133
6	1,426.8 ± 316.6	1,134.2 ± 109.7	0.161
7	1,199.2 ± 41.3	1,314.5 ± 122.7	0.156
8	1,167.5 ± 153.8	1,235.8 ± 109.7	0.512

**Table 4:** The total number of stitches per vessels between the two groups

**(A) Arteries**			
**Trial number**	**Hand (mean ± SD)**	**Robot (mean ± SD)**	***p*** **-value**
1 to 3	2.58 ± 0.51	2.42 ± 0.51	0.436
4 to 8	4.20 ± 0.41	4.50 ± 0.51	0.048
**(B) Veins**			
**Trial number**	**Hand (mean ± SD)**	**Robot (mean ± SD)**	***p*** **-value**
1 to 3	3.00 ± 0.60	3.42 ± 0.51	0.083
4 to 8	4.70 ± 0.47	4.95 ± 0.22	0.041

## Discussion

This study investigates the utility of a robotic microsurgical system in facilitating skill acquisition for supermicrosurgery, specifically in the context of 0.3 mm vessel anastomoses. Despite the technical difficulty associated with such small-caliber targets, the Symani Surgical System enabled progressive improvement in precision, consistency, and procedural control among intermediate-level trainees.

Although the participants had prior experience in conventional microsurgery, robotic assistance allowed them to achieve refined performance within a short series of trials. Notably, SARMS scores were significantly better than the hand-sewn group at some points up to the 7th trial, stitch density was significantly higher after the 4th trial, and patency rates stabilized after the 4th trial. These results indicate that robotic assistance can mitigate the steep learning curve traditionally associated with supermicrosurgical tasks, even at the edge of microsurgical limits.

Consistent with prior findings, robotic-assisted anastomoses demonstrated earlier stabilization in performance quality compared with manual techniques, with comparable patency and SARMS scores achieved within four to five trials. 3 4 6 20 21 22 23 24 Although early robotic procedures were characterized by prolonged operative times, subsequent analysis revealed superior reproducibility and enhanced histological integrity. These observations emphasize the critical role of robotic platforms in advancing technical refinement and ensuring sub-millimetric precision, particularly in the context of complex small-caliber vascular anastomoses. In this trial, initial procedure times were longer in the robotic group for the artery, a pattern previously observed with larger 0.8 mm vessels. 6 However, there was no statistical difference by the 2nd trial although a tendency for longer time was observed, and by the 7th and 8th trials, the time to achieve an anastomosis was negligible between the two groups. Taking into consideration that the total number of stitches performed for each vessel was significantly higher for the robot group, the actual time when considered for per stitch time is less reflecting enhanced dexterity and control. Regarding the vein, the findings showed that it took longer for the hand-sewn group to achieve anastomosis during the first two trials. From the 3rd trial, there was no statistical difference, but the tendency showed longer time needed for the hand-sewn group and ultimately dropped to a negligible difference toward the end of the trials. Similar tendencies were seen with the patency after the anastomosis using the robot. The artery showed 100% leakage-free patency beginning from the 2nd trial and the vein from the 3rd trial while hand sewn took four trials for the artery and five trials for the vein to achieve 100% leakage-free anastomosis. This finding may reflect that the more difficult the vessels are, the faster and accurate the anastomosis may be achieved by the robot. For surgeons who do not routinely perform extreme supermicrosurgery procedures, robotic assistance may facilitate more reliable anastomoses despite infrequent and irregular incidences of supermicrosurgical cases. Overall, these findings suggest that robotic platforms have the potential to enhance both precision and efficiency despite the inexperience.

Robotic assistance has gained increasing traction in lymphatic supermicrosurgery, where stable anastomoses of vessels smaller than 0.3 mm are often required. 13 The findings of this study further validate its utility by demonstrating that consistent, high-quality anastomoses can be achieved within a relatively short learning curve. The integration of advanced robotic platforms with structured assessment frameworks may therefore accelerate the transition to technical proficiency and clinical application.

**Fig. 5:**
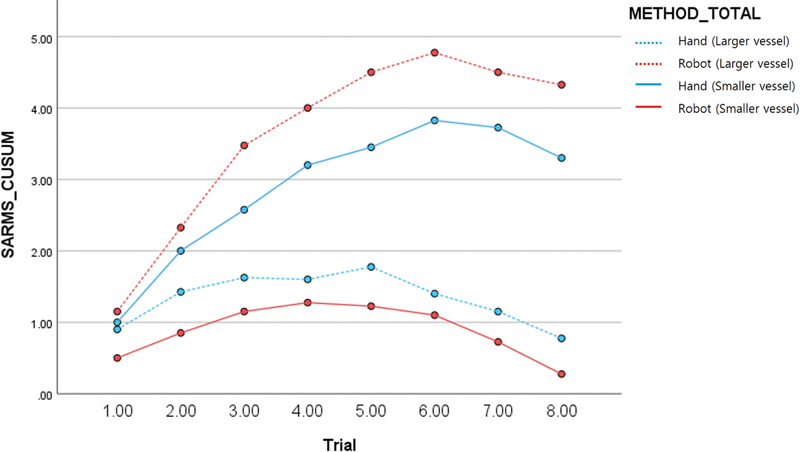
Comparison of cumulative sum (CUSUM) analysis between larger and smaller vessel microanastomosis. Solid lines represent the current study (mean vessel diameter: 0.26 ± 0.05 mm in robot group, 0.26 ± 0.04 mm in hand-sewn group) while dashed lines indicate results from a prior study using larger-caliber vessels (mean: 0.72 ± 0.19 mm in robot, 0.73 ± 0.21 mm in hand). Note the robotic group with the inflection point on the 4th trial and a flatter slope of the graph for the small vessels.

Comparing the data from our previous findings in larger-caliber vessels (0.8 mm), the CUSUM analysis demonstrated several interesting insights ( Fig. 5 ). In larger vessels, while robotic anastomosis required a longer learning phase with the inflection point delayed till the 6th trial, the hand-sewn anastomosis showed a faster inflection point with a flatter curve reflecting shorter learning curve. In contrast, for smaller vessels (0.3 mm), the robotic group exhibited the inflection point at the 4th trial while the hand-sewn group reached inflection point at the 6th trial reflecting a shorter learning curve. When noting the slop of the robotic group, it is even flatter compared against the hand-sewn group for larger vessels indicating rapid adjustment within expected standard of performance. Furthermore, for the hand-sewn approach, it takes a longer learning curve for smaller vessels despite the experience with larger vessels, representing a new form of learning is needed. For the robotic-assisted approach, no additional learning curve is needed, and the skill are transferred from the experience of larger vessels despite the extremely smaller caliber reflecting a muscle memory like effect. Furthermore, the precision and accuracy of the anastomosis were constant with the 0.3 mm vessels as seen with larger vessels when examined under the electron microscope.

Previous preclinical and clinical studies have supported the safety and efficacy of the Symani Surgical System in microsurgery. A preclinical rat model study demonstrated equivalent patency and histological integrity between robotic and manual anastomoses, with no evidence of thrombus formation or distal embolization, highlighting its safety for delicate vascular procedures. 7 These findings were again reproduced in 0.3 mm caliber vessels further validating the robotic approach.

## Conclusion

Robotic-assisted microsurgery holds considerable promise in bridging the transition from fundamental microsurgical skills to advanced supermicrosurgical performance. In the context of 0.3 mm vessel repair, this study demonstrates the feasibility and efficiency of robotic platforms in achieving enhanced technical precision, stability, and procedural reproducibility. These findings provide a robust basis for the continued development and clinical integration of robotic systems in supermicrosurgical practice.
